# Repolarização Ventricular como Ferramenta de Monitoramento da Atividade Elétrica Cardíaca

**DOI:** 10.36660/abc.20201009

**Published:** 2020-11-01

**Affiliations:** 1 Universidade de São Paulo Hospital das Clínicas da Faculdade de Medicina Instituto do Coração (InCor) São PauloSP Brasil Instituto do Coração (InCor) do Hospital das Clínicas da Faculdade de Medicina, Universidade de São Paulo, São Paulo, SP - Brasil

**Keywords:** Pandemia, Coronavírus, Betacoronavírus, COVID-19/complicações, Eletrocardiografia, Intervalo QT, Cardiomiopatia, Arritmias Cardíacas, Embolia, Trombose

O centenário ECG ainda é uma excelente ferramenta para avaliar a atividade elétrica cardíaca. Ao longo das últimas décadas, o ECG tem sido renovado para acompanhar a evolução em outras áreas do conhecimento, como genética, biologia molecular e eletrofisiologia.

Nossa experiência tem demonstrado que, entre o grande arsenal diagnóstico disponível para investigação de cardiopatias, o eletrocardiograma, ferramenta simples, prática, remota e rápida, é capaz de monitorar com precisão a extensão e a gravidade do acometimento cardíaco em diversos cenários.

O intervalo QT e suas variações, muitas décadas após ter sido descrito pela primeira vez, ainda mantêm parâmetros relevantes para indicar se um paciente tem risco de apresentar eventos cardiológicos graves e às vezes fatais.

Em 1856, o primeiro paciente com a síndrome do QT longo foi descrito por Meissner. Embora sua origem genética tenha sido estabelecida em 1901, foi somente em 1991 que Keating demonstrou pela primeira vez a associação de pacientes com síndrome do QT longo e a mutação do braço curto do cromossomo 11. Bazzet, em 1920, descreveu sua fórmula para a correção da frequência cardíaca do intervalo QT.^1^

O surgimento da pandemia da COVID-19 em março de 2020 mostrou uma doença inicialmente com sintomas respiratórios, mas com possível envolvimento de vários outros órgãos devido à sua resposta inflamatória bastante agressiva.

Aproveitando a experiência no tratamento das repercussões cardíacas da COVID-19, os especialistas analisaram os achados eletrocardiográficos durante o período da infecção.

No estudo feito por.. et al.,^2^ publicado nesta edição dos Arquivos Brasileiros de Cardiologia, os autores examinaram as alterações dos intervalos QT, QTc e Tpe (Tpico-Tfinal), e as relações Tpe/QT e Tpe/QTc, todos parâmetros de repolarização ventricular.

O grupo de estudo de 120 pacientes, 90 dos quais infectados com COVID-19 e 30 controles saudáveis pareados por idade e sexo, foi dividido em quatro grupos: I — controles saudáveis e pacientes com COVID-19: II — sem pneumonia, III — com pneumonia leve e IV — com pneumonia grave. Os resultados mostraram que um em cada cinco pacientes com COVID-19 apresentou lesão miocárdica.

O estudo mostrou que nos casos de pneumonia grave existem claras alterações da repolarização ventricular. Apesar de valores de QT praticamente normais, a análise dos parâmetros estudados demonstrou aumento da dispersão da repolarização transmural, que é a etiologia usual das arritmias graves.

As causas mais frequentes de mortalidade cardíaca em pacientes com COVID-19 foram eventos arrítmicos. Os tipos de arritmia foram diversos, com muitos aspectos relevantes. O mecanismo das arritmias não pôde ser caracterizado, mas a literatura relata a presença de fenômenos arrítmicos em 27,8% e de taquicardia ventricular/fibrilação ventricular (TV/FV) em 5,9% dos 187 pacientes estudados por Guo et al.^3^

O mecanismo mais importante das arritmias ventriculares relatado em pacientes com COVID-19 é semelhante ao das arritmias encontradas em pacientes com miocardite aguda. A análise das repercussões da miocardite aguda em outros estudos mostrou aumento dos intervalos QT, QTc e Tpe e das razões Tpe/QT e Tpe/QTc.

No estudo discutido aqui, todas essas medidas aumentaram claramente com a gravidade da doença, como visto nos pacientes com COVID-19 e pneumonia grave.

Confirmando relatos de maior frequência de arritmias em pacientes com níveis aumentados de troponina, o aumento nos níveis de troponina I de alta sensibilidade mostrou uma relação positiva e efetiva com as medidas dos parâmetros QT.

Em um relatório recente^4^, os autores mencionam um estudo ^5^ que categorizou as complicações cardíacas da COVID-19 em cinco tipos:

1. Dano cardíaco (isquemia ou miocardite)
2. Arritmias
3. Novo início ou agravamento de insuficiência cardíaca preexistente
4. Doença tromboembólica
5. Alterações cardíacas induzidas por tratamento médico

Os autores afirmam que “o envolvimento cardíaco em pacientes com COVID-19 se reflete em alterações de ECG como alterações de ST-T, prolongamento de QT, distúrbios de condução e arritmias ventriculares”. Assim, “os pacientes com sintomas cardíacos e anormalidades no ECG devem ser avaliados cuidadosamente para diagnosticar a COVID-19 — complicações cardíacas relacionadas, como miocardite, isquemia miocárdica ou arritmias graves”. ^4^

Nesta pandemia, devemos manter essas suspeitas clínicas mesmo para pacientes que apresentam sintomas ou sinais discretos. Não há dúvida de que a presença de doenças cardiovasculares piora o prognóstico do processo. O vírus não pode ser considerado a causa de todas as complicações cardiovasculares, mas pode piorar ou revelar condições subjacentes precárias.

No artigo aqui discutido^2^, as alterações de repolarização observadas, embora não específicas, requerem investigações adicionais para excluir complicações relacionadas à doença.

A presença de arritmias gerais (16,7%) e malignas (11,5%) também suscitou maior preocupação em casos com comprometimento miocárdico mais grave do que leve.

A comparação do estudo de Haseeb et al., ^4^ e o desta edição dos Arquivos Brasileiros de Cardiologia,^2^ nos leva a concluir que as alterações elétricas detectadas no ECG podem ser relevantes para a tomada de decisão sobre diagnóstico e conduta.

A presença de alterações isquêmicas, prolongamento do intervalo QT, distúrbios de condução elétrica e arritmias no ECG podem ser um grande sinal de alerta para orientar a conduta em caso de comprometimento cardiológico.
