# Supplementary material for: Repolarização Ventricular como Ferramenta de Monitoramento da Atividade Elétrica Cardíaca
Source: Arq Bras Cardiol. 2020 Nov 1;115(5):914–5. [Article in Portuguese] doi: 10.36660/abc.20201009 (PMC8452222; doi:10.36660/abc.20201009)
Supplement: Translation

# Translation: Ventricular Repolarization as a Tool to Monitor Electrical Activity of the Heart

**Affiliations:** 1 São PauloSP Brazil Instituto do Coração (InCor) do Hospital das Clínicas da Faculdade de Medicina, Universidade de São Paulo, São Paulo, SP - Brazil

**Keywords:** Pandemics, Coronavirus, Betacoronavirus, COVID-19/complications, Cardiac Arrhythmias, Embolism and Thrombosis

The centennial ECG is still an excellent tool to assess electrical activity of the heart. ECG has been renovated over the last decades to keep pace with evolution in other areas of knowledge, such as genetics, molecular biology and electrophysiology.

Our experience has demonstrated that, among the large diagnostic arsenal available to investigate heart diseases, the electrocardiogram, this simple, practical, remote and quick tool, is capable of accurately monitoring the extent and severity of cardiac involvement in various scenarios.

QT interval and its variations, many decades after being first reported, still holds relevant parameters to indicate whether a patient is at risk for severe and sometimes fatal cardiological events.

In 1856, the first patient with long QT syndrome was reported by Meissner. Although its genetic origin was established in 1901, it was only in 1991 that Keating first demonstrated the association of patients with long QT syndrome and short arm mutation of chromosome 11. Bazzet, in 1920, reported his formula for heart rate correction of the QT interval.^1^

The emergence of the COVID-19 pandemic in March 2020 showed a disease initially with respiratory symptoms, but with the possible involvement of several other organs due to its very aggressive inflammatory response.

Taking advantage of their experience with treating the COVID-19 cardiac repercussions, experts analyzed electrocardiographic findings during the period of infection.

In the study by Koc et al.^2^ published in this edition of *Arquivos Brasileiros de Cardiologia*, the authors examined the alterations of QT, QTc and Tpe (Ppeak-Tend) intervals, and the Tpe/QT and Tpe/QTc ratios, all of which are parameters of ventricular repolarization.

The study group of 120 patients, 90 of whom infected with COVID-19, and 30 age-and-sex-matched healthy controls, was divided into four groups: I — healthy controls and COVID-19 patients: II — without pneumonia, III — with mild pneumonia, and IV — with severe pneumonia. Results showed that one out of five patients with COVID-19 had myocardial damage.

The study showed that in cases with severe pneumonia there are clear ventricular repolarization alterations. In spite of practically normal QT values, analysis of the parameters studied demonstrated increased dispersion of transmural repolarization, which is the usual etiology of severe arrhythmias.

The most frequent causes of cardiac mortality in patients with COVID-19 were arrhythmic events. The types of arrhythmia were diverse, with many relevant aspects. The mechanism of arrhythmias could not be characterized, but the literature reports the presence of arrhythmic phenomena in 27.8%, and of ventricular tachycardia /ventricular fibrillation (VT/VF) in 5.9% among the 187 patients studied by Guo et al.^3^

The most important mechanism of ventricular arrhythmias reported in patients with COVID-19 is similar to that of arrhythmias found in patients with acute myocarditis. The analysis of acute myocarditis repercussions in other studies showed increased QT, QTc and Tpe intervals, and Tpe/QT and Tpe/QTc ratios.

In the study discussed here, all these measures clearly increased with disease severity, as seen in the COVID-19 patients with severe pneumonia.

Confirming reports of higher frequency of arrhythmias in patients with increased troponin levels, increase in high-sensitivity troponin I levels showed a positive and effective relationship with the measures of QT parameters.

In a recent report,^4^ the authors mention a study^5^ that categorized the cardiac complications of COVID-19 into five types:

1. Cardiac damage (ischemia or myocarditis)
2. Arrhythmias
3. New-onset or worsening of preexisting heart failure
4. Thromboembolic disease
5. Cardiac abnormalities induced by medical treatment

The authors state that “cardiac involvement in COVID-19 patients is reflected in ECG alterations as ST-T alterations, QT prolongation, conduction disorders and ventricular arrhythmias.” Thus, “patients with cardiac symptoms and ECG abnormalities must be carefully assessed in order to diagnose COVID-19-related cardiac complications, such as myocarditis, myocardial ischemia or severe arrhythmias.”^4^

In this pandemic, we must maintain these clinical suspicions even for patients who present with discrete symptoms or signs. There is no doubt that the presence of cardiovascular disease worsens the prognosis of the process. The virus cannot be considered as the cause of all cardiovascular complications, but it can worsen or reveal precarious underlying conditions.

In the article discussed here,^2^ the alterations of repolarization observed, although not specific, call for further investigation to exclude disease-related complications.

The presence of general (16.7%) and malignant (11.5%) arrhythmias also raised greater concern in conditions with a more severe myocardial involvement than with mild involvement.

Comparison of the study by Haseeb et al.^4^ and the one in this edition of *Arquivos Brasileiros de Cardiologia*^2^ leads us to conclude that electrical alterations detected in the ECG can be relevant to make a decision about diagnosis and management.

The presence of ischemic alterations, QT prolongation, electrical conduction disorders and arrhythmias in the ECG can be a big warning sign to guide management in case of cardiological involvement.
